# Your words went straight to my heart: the role of emotional prototypicality in the recognition of emotion-label words

**DOI:** 10.1007/s00426-022-01723-6

**Published:** 2022-09-03

**Authors:** Juan Haro, Rocío Calvillo, Claudia Poch, José Antonio Hinojosa, Pilar Ferré

**Affiliations:** 1 Departament de Psicologia and CRAMC, Universitat Rovira i Virgili, Carretera de Valls, s.n., 43007 Tarragona, Spain; 2 Departamento de Psicología Experimental, Procesos Cognitivos y Logopedia, Universidad Complutense de Madrid, Madrid, Spain; 3 Departamento de Educación, Universidad de Nebrija, Madrid, Spain; 4 Instituto Pluridisciplinar, Universidad Complutense de Madrid, Madrid, Spain; 5 Centro de Investigación Nebrija en Cognición (CINC), Universidad Nebrija, Madrid, Spain

## Abstract

Emotional words differ in how they acquire their emotional charge. There is a relevant distinction between emotion-label words (those that directly name an emotion, e.g., “joy” or “sadness”) and emotion-laden words (those that do not name an emotion, but can provoke it, e.g., “party” or “death”). In this work, we focused on emotion-label words. These words vary in their emotional prototypicality, which indicates the extent to which the word refers to an emotion. We conducted two lexical decision experiments to examine the role played by emotional prototypicality in the recognition of emotion-label words. The results showed that emotional prototypicality has a facilitative effect in word recognition. Emotional prototypicality would ease conceptual access, thus facilitating the retrieval of emotional content during word recognition. In addition to the theoretical implications, the evidence gathered in this study also highlights the need to consider emotional prototypicality in the selection of emotion-label words in future studies.

## Introduction

The interplay between cognition and emotion has been intensively studied in recent decades. This topic has been examined in a variety of cognitive domains, including language, in general, and word recognition, in particular. Most studies in the field have compared the processing of affective (emotional) and non-affective (neutral) words. A lot of the research has relied on emotional valence and emotional arousal to characterize emotional words, as many authors have found that these dimensions are the most relevant for describing the human affective space (e.g., Bradley & Lang, 1999). Emotional valence refers to the affective value of a word, which can range from very negative/aversive to very positive/appealing. For instance, the word “gun” is a negatively valenced word (i.e., a negative word), whereas the word “friendship” is a positively valenced one (i.e., a positive word). Emotional arousal, on the other hand, indicates the degree of activation associated with a word, from very calming/relaxing to very activating/exciting. For instance, the word “boredom” has a low arousal while “explosion” has a high arousal. There is substantial evidence, mostly obtained from lexical decision task (LDT) studies, that both valence and arousal influence word recognition (e.g., Kousta et al., 2009, Kuperman et al., 2014, Rodríguez-Ferreiro & Davies, 2018, see also Hinojosa et al., 2020, for a review), with positive (e.g., Vinson et al., 2014) and highly arousing words (e.g., Vieitez et al., 2021) being recognized faster than neutral and low arousing words. These effects have been shown to be modulated by several lexico-semantic variables, such as lexical frequency (e.g., Méndez-Bertolo et al., 2011; Palazova et al., 2011; Scott et al., 2009) and concreteness (e.g., Palazova et al., 2013; Ponari et al., 2018).

A variable that may be relevant in this field of research is the relationship between the emotional word and the emotion it conveys (e.g., Altarriba, 2006; Pavlenko, 2008). Not all emotional words acquire their affective charge in the same way. Some of them, the so-called emotion-label words (EM henceforth), name an emotion directly. This category includes words like “joy”, “anger” and “sadness”. Emotion-laden words (EL henceforth), in contrast, acquire their emotional charge through indirect links with other concepts or words that denote emotions. For instance, the word “death” is a negative word that does not label any emotion, and that probably receives its affective charge from its connections to “sadness” and other words referring to related emotions. The few studies that have examined the distinction between EM and EL words to date have shown differences between the processing of these two types of words. Indeed, EM words elicited a greater repetition blindness effect than EL words in a rapid serial visual presentation task (Knickerbocker & Altarriba, 2013). In addition, EM words were recognized faster and produced a greater priming effect than EL words in a primed lexical decision task (Kazanas & Altarriba, 2015). Neurophysiological evidence coming from event-related potential (ERP) studies also supports the distinction between EM and EL words (Wang et al., 2019; Wu et al., 2021; Zhang et al., 2017). These studies report differences between EM and EL words in the N170 (Zhang et al., 2017) and P2 (Wang et al., 2019) components, suggesting that these two types of words are differentiated early in word processing. A possible reason for the differences in processing between EM and EL words may be the more salient emotional content in EM words than in EL words. This would be a consequence of direct conceptual links between EM words and the core emotions they convey (Knickerbocker & Altarriba, 2013).

This study focuses on the processing of EM words and the role of an unexplored variable, emotional prototypicality. According to the prototype theory of emotion (Niedenthal et al., 2004; Shaver et al., 1987; Zammuner, 1998), EM words vary in the extent to which they refer to an emotion (e.g., Fehr & Russell, 1984). This idea, inspired by Rosch’s work (1978) on categorical prototypicality, means that emotional concepts are not defined in terms of a set of necessary and sufficient attributes, but rather vary in the degree to which they express an emotion. Therefore, some words would be better exemplars (i.e., more representative) of the semantic category of emotion than others and would therefore convey an emotional meaning more strongly. From this perspective, normative data on emotional prototypicality have been collected in different languages (e.g., Alonso-Arbiol et al., 2006; Galati et al., 2008; Niedenthal et al., 2004; Pérez-Sánchez et al., 2021; Shaver et al., 1987, 2001; Zammuner, 1998). These studies have relied on three different tasks: (1) Free listing of emotion terms, where participants are asked to name all emotions that come to mind in a short period of time, (2) emotional prototypicality rating task, where participants are asked to rate in a numerical scale the extent to which a set of potential EM words refer to an emotion (i.e., how good each word is perceived as an emotion-label word) (e.g., Alonso-Arbiol et al., 2006; Galati et al., 2008; Niedenthal et al., 2004; Pérez-Sánchez et al., 2021; Shaver et al., 1987, 2001; Zammuner, 1998) and, (3) categorization task, where participants have to determine if a set of potential EM words refer to an emotion or not while reaction times are recorded (e.g., Niedenthal et al., 2004). The results of these tasks clearly show that some EM words come more easily to mind, are categorized quicker as emotion terms, and are given higher emotional prototypicality ratings than others. That is, speakers perceive some emotion terms (e.g., “happiness”) as more prototypical than others (e.g., “boredom”).

Some of the above-mentioned studies have examined the predictive capacity of several variables on emotional prototypicality ratings. The most representative exemplars of the category of emotions are words that denote an intense affective experience (regardless of its positive or negative sign) (Niedenthal et al., 2004; Pérez-Sánchez et al., 2021; Zammuner, 1998). This intense affective experience cannot be explained exclusively in terms of either valence or arousal because both factors contribute (Pérez-Sánchez et al., 2021), as well as the duration of the experience (i.e., words of high emotional prototypicality denote brief affective experiences, Zammuner, 1998).

Another interest of this line of research has been to define the structure of the emotion lexicon. To that end, researchers have applied hierarchical cluster analyses to people’s similarity judgments between pairs of words (Alonso-Arbiol et al., 2006; Galati et al., 2008; Shaver et al., 1987, 2001). These studies have shown that emotion categories, like other categories, can be organized hierarchically, with supraordinate, subordinate and basic levels. At the top of the hierarchy, there is a binary distinction between positive and negative emotions. Then, below the superordinate level, there are a few basic emotions (love, happiness, anger, sadness, and fear). Finally, below this level, there are many other emotions, such as relief or disappointment. Like in other domains (e.g., furniture, sports, animals, etc.), basic exemplars have a higher emotional prototypicality than subordinate exemplars (Pérez-Sánchez et al., 2021). Similarly, there is a processing advantage for basic exemplars: people are faster when categorizing basic-level emotions and children learn the names for those emotions earlier during language acquisition (see Edelstein & Shaver, 2007, for a review).

Word prototypicality in general has been related to how quickly a word may be retrieved from the mental lexicon, which is known as the typicality effect (Jerger & Damian, 2005). Indeed, more typical exemplars show an advantage in processing with respect to less typical exemplars in semantic tasks such as category verification (Jerger & Damian, 2005), semantic fluency (Crowe & Prescott, 2003), animacy decision (Räling et al., 2017) and category naming (Hampton, 1995). The typicality effect has been observed also in tasks involving both lexical and semantic processes like reading (Garrod & Sanford, 1977), sentence production (Kelly et al., 1986) and picture naming (Holmes & Ellis, 2006. This is considered a semantic effect (Woollams, 2012) that indicates ease of conceptual access (see Folstein & Diecuc, 2019, for a review). The typicality effect has been mostly demonstrated with concrete categories, such as furniture, vegetables, animals, or sports, among others. Of relevance here are several studies conducted with the semantic category of “emotions”, which have also shown the effect. For example, Fehr et al. (1982) asked their participants to perform a category verification task. Responses were faster for words of high emotional prototypicality than for words of low emotional prototypicality (see Fehr & Russell, 1984, 1991; Russell & Fehr, 1994; Niedenthal et al., 2004; Zammuner, 1988, for similar results). In another study, Hnazaee and Van Hulle (2017) examined the modulation by category typicality of an event-related potentials (ERP) component (N400), related to semantic integration (Kutas et al., 2011). In a semantic priming experiment, participants had to decide whether the second word of a pair was a member of the category labeled by the prime word. Crucially, the prototypicality of the second word with respect to that category was manipulated, and there were both concrete and abstract pairs of words, among them words referred to emotions. The results showed a word typicality effect on N400 independently of the abstractness of the words. The results of these studies suggest that emotion categories behave similarly to other categories in terms of the role of prototypicality.

Taking all the above into consideration, emotional prototypicality seems to be a good measure of the representativeness of words as members of the EM category. The main aim of this study is precisely to examine the role of this variable in word recognition using a LDT. Previous studies have demonstrated that emotional content facilitates word recognition. Our rationale is that, if prototypicality facilitates conceptual access, EM words of high emotional prototypicality, which denote an intense affective experience, should be recognized faster than EM words of low emotional prototypicality. We conducted two LDT experiments, in which we examined whether the emotional prototypicality of EM words facilitates word recognition and whether the effect of this variable is independent of that of other affective (e.g., emotional valence and emotional arousal), lexical (e.g., word frequency) and semantic (e.g., concreteness) variables that have been shown to influence word recognition (see Hinojosa et al., 2020, for a review).

## Experiment 1

### Method

#### Participants

Fifty-nine participants completed the experiment.^1^ They had a mean age of 22.73 years (SD = 6.90; range = 19—60). Fifty-two participants were females and 7 of them were males. All participants were students from the Rovira i Virgili University (Tarragona, Spain) or the Complutense University of Madrid (Madrid, Spain), and they received a bonus of academic credits in exchange for their participation. All of them signed an informed consent form prior to the study.

#### Materials

We randomly selected 100 Spanish words, all of them nouns, with different degrees of emotional prototypicality, from the database of Pérez-Sánchez et al. (2021). The scale of emotional prototypicality in that study ranges from 1 (This word does not refer to an emotion) to 5 (This word clearly refers to an emotion). The words chosen had values ranging from 1.27 to 4.90 in emotional prototypicality, with a mean score of 2.94 and a standard deviation of 0.83. There were 52 words of low prototypicality (prototypicality < 3; e.g., *alerta,* “alert”) and 48 words of high prototypicality (prototypicality > 3; e.g., *miedo,* “fear”). According to the valence categorization criteria used in previous studies (e.g., Huete-Pérez et al., 2020), forty-nine of these words had a negative valence (valence < 4), 14 had a neutral valence (4 ≤ valence ≤ 6), and 37 had a positive valence (valence > 6). The descriptive statistics of the variables included in the study are shown in Table 1. We also selected 100 filler words, which were pairwise matched with the experimental stimuli on log word frequency, number of letters, number of neighbors, concreteness, and age of acquisition (all *p*s > 0.492). All the fillers were neutral (non-emotional) words (4 ≤ valence ≤ 6; e.g., *fracción*, “glare”). Finally, we used the Wuggy software to generate 200 nonwords from the 200 words (Keuleers & Brysbaert, 2010). The nonwords were pronounceable and orthographically legal in Spanish (e.g., *planor*, *ingrama*, *rosto*), and they were matched to the words in number of letters and syllables, subsyllabic structures, and transition frequencies. In summary, 400 stimuli were included in the experiment: 100 EM words varying in emotional prototypicality (i.e., the critical stimuli), 100 non-emotional (neutral) filler words, and 200 nonwords.

**Table 1:** Descriptive statistics of the EM words (the critical stimuli) included in the experiments

	*M*	SD	Minimum	Maximum
Log word frequency	1.26	0.42	0.35	2.29
Number of letters	8.14	2.02	4.00	13.00
Number of neighbors	1.77	2.61	0.00	15.00
Concreteness	4.07	0.61	2.40	6.24
Age of acquisition	8.10	1.45	3.60	10.36
Emotional prototypicality	2.94	0.83	1.27	4.90
Emotional valence	4.74	2.46	1.50	8.60
Emotional arousal	5.88	1.67	1.50	8.25

The ratings of emotional variables (emotional valence and arousal), concreteness, and age of acquisition were obtained from Stadthagen-Gonzalez et al. (2017), Pérez-Sánchez et al. (2021), Alonso et al. (2015), EsPAL (Duchon et al., 2013), and Hinojosa et al. (2016). We used EmoFinder (Fraga et al., 2018) to retrieve the ratings from some of the databases. In addition, we obtained the logarithm of word frequency (log word frequency) and the number of neighbors of these words from EsPAL (Duchon et al., 2013)

#### Procedure

All the stimuli were presented in a lexical decision task, which was administered online. The JsPsych software (de Leeuw, 2015) was used to present the stimuli and record the responses. Participants were presented with 400 trials in a totally randomized order. Each trial began with the presentation of a fixation point (“ + ”) in the center of the screen for 500 ms. The fixation point was then replaced by a string of letters (a word or a nonword) and the participants had to indicate whether the string corresponded to a word or not. Participants were asked to use the keyboard for responding, with one key to answer “NO” and another key to answer “YES”. The string of letters disappeared after the response or after 3000 ms. Participants received a feedback message after they had responded: “CORRECT”, “ERROR” or “NO RESPONSE” (if 3000 ms had elapsed and no response had been received). The feedback message was displayed on the screen for 1000 ms. After the feedback, the screen remained blank for 750 ms, and then the next trial was automatically presented. Before starting the experiment, participants underwent a practice phase consisting of 16 trials, in which they were presented with 8 words and 8 nonwords not included in the experimental phase. Participants were allowed to have a short break in the middle of the experiment. There was no time limit for the break; instead, participants could decide when to continue with the experiment. In any case, no participant spent more than 5 min in the break. The total duration of the experiment was around 20 min.

#### Data analyses

We obtained 11,800 RTs in total. RTs below 300 ms and above 2,000 ms were removed (*n* = 100), as were RTs that were 2 SDs above or below each participant’s mean RT (*n* = 311). The data from one participant with 45% errors and the data from one word with 70.69% errors (*fulgor*, “glare” in English) were rejected. Due to the small percentage of errors committed (mean = 5.16%, range = 0.25–13.25%), the errors were not analyzed, and the RTs corresponding to these were removed (*n* = 553). We removed a total of 1222 data points (10.36% of the total), keeping 10,578 data points for the analyses.

We used RStudio version 1.1.463 (RStudio Team, 2018) running R version 4.0.2 (R Core Team, 2020) to conduct the analyses. We analyzed the RT data by means of linear mixed-effect models (e.g., Baayen, 2008; Baayen et al., 2008), using the lme4 package from R (Bates et al., 2019). Different linear models were generated to independently examine the effect of emotional prototypicality, the effect of affective variables (emotional valence and emotional arousal), and the interaction between them on inverse RTs (− 1000/*RT*). Two sets of models were created. The first set included only the EM words, with the aim of examining the contribution of emotional prototypicality, and that of valence and arousal, in their recognition. The second set included all the words (i.e., the EM words and the fillers) with the aim of examining whether the usual effects of affective variables (i.e., valence and arousal) would be observed in these stimuli. In this second set of models, emotional prototypicality was not included because filler words had no emotional prototypicality scores (they were neutral words). All variables and interactions were introduced as fixed effects, and we also added some lexical and semantic variables (see Table 1) into each model as covariates to control for their potential effect on RTs. Each model included random intercepts for participants and words. Although we tried to follow a maximal random-effects structure (Barr et al., 2013), it was not possible to include any random slope to the models since this resulted in singular fit or convergence error. We also report the results of the *t* test analyses for the coefficient estimates of each fixed effect. To this end we used Satterthwaite’s approximations to the degrees of freedom of the denominator (*p* values were estimated using the lmerTest package, Kuznetsova et al., 2019).

### Results and discussion

The first set of models was restricted to EM words (i.e., the critical words) and examined the effect of emotional prototypicality, and the interaction between this variable and the rest of the semantic, affective, and lexical variables (see Table 2 for the results and formula of the final model). We started with the most complex model based on the combination of emotional variables (i.e., that including the triple interaction between valence x arousal x emotional prototypicality). We gradually reduced the complexity of the model by removing interactions at each step: first the interaction of third order and then those of second order. After each step, we compared the model of higher and lower complexity by means of likelihood-ratio chi-squared tests (using the R ANOVA function); if the comparison was not significant, we kept the model of lower complexity. Following this process we reached a model with no interactions, only main effects, which was compared with a model where emotional prototypicality was not included. Thus, the final model for the analysis of EM words included the main effects of the emotional variables, as well as the effects of the lexical and semantic variables that were introduced as covariates. The interaction between prototypicality and the lexical and semantic variables was also examined in this model, yielding a non-significant result in all cases.

**Table 2:** Results of the linear mixed effects model used to examine the effects of emotional prototypicality on the RT of EM words

Predictors	Estimate	SE	*t*	*p*
Intercept	− 1.67	0.12	− 13.38	< 0.001
Emotional valence	− 0.01	0.00	− 1.55	0.120
Emotional arousal	0.00	0.01	0.64	0.520
Emotional prototypicality	− 0.03	0.01	− 2.89	0.004
Log. word frequency	− 0.11	0.02	− 4.78	< 0.001
Number of letters	0.03	0.00	5.10	< 0.001
Number of neighbors	0.01	0.00	2.41	0.016
Concreteness	− 0.01	0.01	− 0.90	0.367
Age of acquisition	0.02	0.01	2.53	0.012
Trial order	0.00	0.00	5.82	< 0.001

Formula of the final model: − 1000/*rt* ~ valence + arousal + prototypicality + frequency + number_of_letters + number_of_neighbors + concreteness + age_of_acquisition + trial_order + (1 | participant) + (1 | word)

We found that emotional prototypicality had a facilitating effect, *χ*^2^(1) = 8.69, *p* = 0.003, which indicates that high prototypicality words were recognized faster than low prototypicality words; indeed, the mean *RT* for words with a prototypicality rating above 3 was 676.81 (SD = 201.62), while the mean *RT* for words with a prototypicality rating below 3 was 688.29 (SD = 203.34). The model that included emotional prototypicality was superior (AIC = 1385.2, BIC = 1471.0, variance explained by fixed effects = 5.30%) to the one that did not include it (AIC = 1391.9, BIC = 1471.1, variance explained by fixed effects = 4.98%). There was not any significant interaction between prototypicality and the other variables (all *p*s > 0.05). Moreover, a facilitating effect of word frequency was observed, as well as an inhibitory effect of number of letters, age of acquisition, number of neighbors, and trial order (all *p*s < 0.05).

In the second set of models, we examined the effects of emotional valence and arousal, as well as their interaction with the rest of the semantic and lexical variables, using the data from all the words presented in the experiment (i.e., the EM words and the neutral filler words, *n* = 200; see Table 3 for the results and formula of the final model). We followed the same procedure as described above for the EM words analyses, but without including emotional prototypicality (i.e., the most complex model included the interaction between valence and arousal).

**Table 3:** Results of the linear mixed effects model used to examine the effects of emotional variables (emotional valence and arousal) on the *RT* of all the words

Predictors	Estimate	SE	*t*	*p*
Intercept	− 1.39	0.13	− 10.47	< 0.001
Emotional valence	− 0.05	0.02	− 3.51	< 0.001
Emotional arousal	− 0.05	0.01	− 3.46	0.001
Emotional valence * emotional arousal	0.01	0.00	3.07	0.002
Log. word frequency	− 0.10	0.02	− 6.03	< 0.001
Number of letters	0.01	0.00	4.19	< 0.001
Number of neighbors	0.00	0.00	0.76	0.446
Concreteness	0.01	0.01	0.95	0.344
Age of acquisition	0.02	0.00	3.49	< 0.001
Trial order	0.00	0.00	8.95	< 0.001

Formula of the final model: -1000/*rt* ~ valence*arousal + frequency + number_of_letters + number_of_neighbors + concreteness + age_of_acquisition + trial_order + (1 | participant) + (1 | word)

The results showed a significant facilitating effect of valence, with negatively valenced words being recognized more slowly than positively valenced words (*p* < 0.001). The effect of arousal also reached significance, suggesting that the higher the arousal, the faster the word recognition (*p* = 0.001). The main effects of valence and arousal were qualified by the interaction between the two variables, *χ*^2^(1) = 9.61, *p* = 0.002, indicating that a negative valence slows down the response to low arousal words, but not to high arousal words. On the other hand, a facilitating effect of word frequency was observed, as well as an inhibitory effect of number of letters, age of acquisition, and trial order (all *p*s < 0.01).

The most relevant finding of this experiment is the facilitating effect of emotional prototypicality on the lexical decision task, reported here for the first time. One possible mechanism explaining this facilitation is the easier conceptual access (i.e., easier access to affective-semantic information) for EM words of high emotional prototypicality. Regarding the other affective variables examined here, the effects of emotional valence and arousal found in the analyses that included all the words are in line with previous LDT studies (see Hinojosa et al., 2020, for a review). It is important to note, however, that these effects were not found in the analysis restricted to EM words. This result will be discussed in depth in the General Discussion. Finally, the effects observed for the lexical and semantic variables included in the models are compatible with previous evidence (see González-Nosti et al., 2014, for similar effects with Spanish words in LDT).

Before drawing conclusions on the role played by prototypicality in word recognition, there is a possible confounding factor that needs to be discarded. Specifically, all the affectively charged (emotional) words in this experiment were EM words, since filler words were all neutral in valence. Therefore, emotional prototypicality might be very prominent for participants and affect word recognition for this reason. To rule out this possibility, we conducted a second experiment in which we also included EL words (i.e., words like “death”, which are affectively charged, but do not name any emotion) as fillers. Consequently, emotional prototypicality would not be such a prominent variable, since not all emotional words presented to participants would refer to emotions.

## Experiment 2

### Method

#### Participants

Sixty-three participants completed the experiment. As in Experiment 1, all of them were native speakers of Spanish. They had a mean age of 22.95 years (SD = 5.42; range = 19–46). Fifty of them were females and thirteen were males. They were students from the Rovira i Virgili University (Tarragona, Spain) and the Complutense University of Madrid (Madrid, Spain), who received a bonus of academic credits in exchange for their participation. All of them signed an informed consent form before starting the experiment.

#### Materials

We used the same 100 critical EM words as in Experiment 1. The difference was that, in Experiment 2, half of the fillers (i.e., 50 words) were emotional words, specifically, EL words (i.e., words that get their emotional charge from indirect links to concepts or words that denote emotions) with either a positive (e.g., *caricia,* “caress”) or negative valence (e.g., *corrupción*, “corruption”), and the other half (i.e., the other 50 fillers) were neutral words (randomly selected from fillers of Experiment 1). Therefore, there were twice as many EM words (*n* = 100) as EL words (*n* = 50) in the experiment. As in Experiment 1, filler words were pairwise matched with the experimental stimuli in log word frequency, number of letters, number of neighbors, concreteness, and age of acquisition (all *p*s > 0.322). To sum up, 400 stimuli were included in the experiment: 100 EM words varying in emotional prototypicality (i.e., the critical stimuli), 50 EL words and 50 neutral words (i.e., the 100 filler words), and 200 nonwords.

#### Procedure

The procedure was identical to that of Experiment 1.

#### Data analyses

We performed the same analyses as in Experiment 1. We obtained 12,600 RTs in total. RTs below 300 ms and above 2,000 ms (*n* = 83) were removed, as well as RTs that were 2 *SD*s above or below the mean RT of each participant (*n* = 332). The data from one participant with 16% errors were rejected. RTs corresponding to errors (*n* = 576) were not included in the analyses. As in Experiment 1, errors were not analyzed because there was only a small number of them (mean = 4.83%; range = 0.5–12.5%). In total we removed 1191 data points (9.45% of the total), keeping 11,409 data points for the analyses.

### Results and discussion

As in Experiment 1, the first set of models was restricted to EM words and examined the effect of emotional prototypicality and the interaction between this variable and the rest of the variables. The results and formula of the final model are shown in Table 4.

**Table 4:** Results of the linear mixed effects model used to examine the effects of emotional prototypicality on the RT of EM words

Predictors	Estimate	SE	*t*	*p*
Intercept	− 1.55	0.12	− 12.65	< 0.001
Emotional valence	− 0.00	0.00	− 0.22	0.826
Emotional arousal	0.01	0.01	0.93	0.353
Emotional prototypicality	− 0.03	0.01	− 3.17	0.002
Log. word frequency	− 0.11	0.02	− 5.27	< 0.001
Number of letters	0.01	0.00	2.58	0.010
Number of neighbors	0.00	0.00	1.15	0.252
Concreteness	− 0.02	0.01	− 1.55	0.120
Age of acquisition	0.01	0.01	2.24	0.025
Trial order	0.00	0.00	1.95	0.052

Formula of the final model: − 1000/*rt* ~ valence + arousal + prototypicality + frequency + number_of_letters + number_of_neighbors + concreteness + age_of_acquisition + trial_order + (1 | participant) + (1 | word)

Similar to Experiment 1, there was a facilitating effect of emotional prototypicality on the RTs, *χ*^2^(1) = 10.39, *p* = 0.001. High prototypicality words were recognized faster than low prototypicality words; indeed, the mean *RT* for words with a prototypicality rating above 3 was 656.96 (SD = 202.23), whereas the mean *RT* for words with a prototypicality rating below 3 was 675.95 (SD = 208.74). The model including emotional prototypicality provided a better fit to the data (AIC = 2129.6, BIC = 2216.2, variance explained by fixed effects = 2.98%) than the model that did not include it (AIC = 2137.9, BIC = 2217.9, variance explained by fixed effects = 2.63%). No interaction was found between prototypicality and the rest of the variables (all *p*s > 0.05). A facilitating effect of log word frequency was also observed (*p* < 0.001). In addition, an inhibitory effect was found for the number of letters and age of acquisition (both *p*s < 0.05).

In another set of models, we examined the effects of emotional valence and arousal, as well as their interaction with the other variables (see Table 5 for the results and formula of the final model). In these models, we considered the data from all the words presented in the experiment, including filler words (*n* = 200). As in Experiment 1, emotional prototypicality was not included in these models.

**Table 5:** Results of the linear mixed effects model used to examine the effects of emotional variables (emotional valence and arousal) on the *RT* of all the words

Predictors	Estimate	SE	*t*	*p*
Intercept	− 1.43	0.14	− 10.35	< 0.001
Emotional valence	− 0.05	0.02	− 2.88	0.004
Emotional arousal	− 0.04	0.01	− 2.94	0.003
Emotional valence * emotional arousal	0.01	0.00	2.93	0.003
Log. word frequency	− 0.10	0.02	− 6.26	< 0.001
Number of letters	0.01	0.00	3.07	0.002
Number of neighbors	0.00	0.00	0.63	0.529
Concreteness	0.01	0.01	0.54	0.589
Age of acquisition	0.02	0.00	3.24	0.001
Trial order	0.00	0.00	3.16	0.002

Formula of the final model: − 1000/*rt* ~ valence*arousal + frequency + number_of_letters + number_of_neighbors + concreteness + age_of_acquisition + trial_order + (1 | participant) + (1 | word)

As in Experiment 1, the results showed that negatively valenced words were recognized more slowly than positively valenced words (*p* = 0.004), and that arousal had a facilitating effect on *RT*s (*p* = 0.003). Again, the main effects of valence and arousal were qualified by the interaction between the two variables, *χ*^2^(1) = 8.77, *p* = 0.003, showing that a negative valence delayed the response to low arousal words, but had no effect on high arousal words. In addition, a facilitating effect of word frequency was observed, as well as an inhibitory effect of number of letters, age of acquisition, and trial order (all *p*s < 0.01).

The main finding of this experiment was the same as in Experiment 1: a facilitating effect of emotional prototypicality on the recognition of EM words. Importantly, the effect was also obtained when there were emotional (EL) words among the filler words, which suggests that the results of Experiment 1 were not due to the high salience of prototypicality in the experimental list. In addition, the same effect of the different lexical and semantic variables examined was obtained in this experiment. Finally, as in Experiment 1, no effect of emotional valence or arousal was found for the EM words, which contrasts with the effect of these variables in the analysis that included all the words.

## General discussion

The present study examined for the first time the role of emotional prototypicality in EM word recognition. Convergent evidence from two LDT experiments suggests that this variable facilitates EM word recognition, an effect which cannot be attributed to other variables that have been shown to influence LDT. Importantly, the results of the two experiments are consistent, both in terms of the effects of emotional prototypicality on the recognition of EM words (which have been shown to be independent of the type of filler words used), and the effects of affective variables (emotional valence and arousal) when all words are considered.

This study shows that emotional prototypicality plays a significant role in the processing of EM words. One plausible explanation of this effect is the one proposed for the typicality effect observed in several lexico-semantic tasks for distinct categories. That is, ease of conceptual access (Jerger & Damian, 2005). The category of emotions exhibits properties like those of other categories, namely, a hierarchical organization (Alonso-Arbiol et al., 2006; Galati et al., 2008; Shaver et al., 1987, 2001), and a similar pattern of findings in categorization tasks (Fehr et al., 1982; Niedenthal et al., 2004; Zammuner, 1998) and in some experimental paradigms like the semantic priming paradigm (Hnazaee & Van Hulle, 2017). Therefore, like in other categories, conceptual access (i.e., the access to semantic-affective information) would be faster for EM words of high emotional prototypicality than for EM words of low emotional prototypicality. Importantly, the modulating role of semantic and affective variables (e.g., concreteness, semantic ambiguity, valence, and arousal) in the LDT has been firmly established in past research (see Pexman, 2020, and Hinojosa et al., 2020, for reviews). Those semantic and affective properties contribute to the semantic richness of words (Pexman, 2020). Semantic richness may modulate word recognition in the lexical decision task through cascaded interactive activation mechanisms that allow feedback from semantic to lexical representations (Pexman, 2012; Yap et al., 2015). Therefore, if conceptual access is faster for EM words of high emotional prototypicality, there is more room for affective information to influence word recognition through those feedback mechanisms. This issue should be addressed in future research using measures that allow us to examine the online processing (e.g., millisecond-by-millisecond) of words (e.g., ERPs). They would provide valuable information about a possible earlier onset of emotional effects for words of high emotional prototypicality.

Apart from that, the effect of emotional prototypicality might be related to the finding that EM words are processed distinctly from EL words in several experimental tasks (e.g., Kazanas & Altarriba, 2015; Knickerbocker & Altarriba, 2013). Indeed, previous research comparing EM and EL words showed a facilitation for EM words (e.g., Kazanas & Altarriba, 2015). Presumably, these studies included the most representative EM words in their experimental materials. Although they did not rely on published normative studies, the selected EM words probably have high emotional prototypicality values. In view of the facilitation of emotional prototypicality in EM word recognition found in the present study, this may have contributed to previous reports of an advantage for EM words over EL words. In contrast, there are also reports of lack of differences in processing between EM and EL words (e.g., Martin & Altarriba, 2017; Vinson et al, 2014). These studies might have included EM words with lower emotional prototypicality.

A last result that deserves to be commented is the lack of valence effects on the processing of EM words. We can speculate about some possible reasons for this null result. The first reason may be related to methodological issues; specifically, we might have selected EM words that do not have valence ratings that are extreme enough (given that the main objective of this study was not to examine this variable, but rather to study emotional prototypicality), or EM words that do not have enough variability in their valence ratings. The stimulus set of EM words of the present study included more negative than positive words (49 vs. 37), and only 14 neutral words. It should be noted that this selection reflects the valence distribution in EM words: there are more negative words naming emotions than positive words, and there are only a small number of neutral EM words. The second reason may be related to the time-course of the affective modulation of word recognition. It may be that the effect of emotional prototypicality led to a strong acceleration of the emotional evaluation process. This would cause the effects of emotional valence to occur significantly before the participant’s response, without affecting it. Future ERP studies may shed light on this issue.

In conclusion, the present study provides the very first evidence that emotional prototypicality influences the recognition of EM words. Emotional prototypicality would ease conceptual access, thus facilitating affective content to influence word processing. These findings not only have theoretical implications for our understanding of emotional word processing, but also methodological implications, since they suggest that future studies should consider this variable in the selection of EM words. Finally, future research should be conducted to examine at what stage of word processing does emotional prototypicality have an effect, as well as explore in greater depth its relationship with other emotional variables, in particular, emotional valence and arousal.

## Data Availability

An excel file with the values of the stimuli in several psycholinguistic variables (stimuli.xlsx), as well as the script and csv files used in the analyses (“data” directory) have been uploaded to an OSF repository. Web link: https://osf.io/975qr/files/.

## Footnotes

1 To test the statistical power of Experiments 1 and 2, we performed an a posteriori estimation using the SIMR package of R (Green & MacLeod, 2016). The simulation results showed that the statistical power of the experiments was above 80%.
